# Maternal hypertensive traits and adverse outcome in pregnancy: a Mendelian randomization study

**DOI:** 10.1097/HJH.0000000000003486

**Published:** 2023-07-05

**Authors:** Maddalena Ardissino, Rohin K. Reddy, Eric A.W. Slob, Jack Griffiths, Joanna Girling, Fu Siong Ng

**Affiliations:** a National Heart and Lung Institute, Imperial College London, London; b Royal Papworth Hospital, Cambridge Biomedical Campus; c MRC Biostatistics Unit, School of Clinical Medicine, University of Cambridge, Cambridge, UK; d Department of Applied Economics, Erasmus School of Economics; e Erasmus University Rotterdam Institute for Behavior and Biology, Erasmus University Rotterdam, Rotterdam, The Netherlands; f Royal Brompton Hospital, Guy's and St Thomas’ NHS Foundation Trust; g West Middlesex Hospital, Chelsea and Westminster Hospital NHS Foundation Trust, London, UK

**Keywords:** genetics, hemorrhage, hypertension, Mendelian randomization, placental abruption, preeclampsia, pregnancy, preterm birth

## Abstract

**Introduction:**

Hypertensive disorders of pregnancy are associated with adverse feto-maternal outcomes. Existing evidence is mostly limited to observational studies, which are liable to confounding and bias. This study investigated the causal relevance of component hypertensive indices on multiple adverse pregnancy outcomes using Mendelian randomization.

**Methods:**

Uncorrelated (*r*^2^ < 0.001) genome-wide significant (*P* < 5 × 10^−8^) single-nucleotide polymorphisms associated with SBP, DBP and pulse pressure (PP) were selected as instrumental variables. Genetic association estimates for outcomes of preeclampsia or eclampsia, preterm birth, placental abruption and hemorrhage in early pregnancy were extracted from summary statistics of genome-wide association studies in the FinnGen cohort. Two-sample, inverse-variance weighted Mendelian randomization formed the primary analysis method. Odds ratios (OR) are presented per-10 mmHg higher genetically predicted hypertensive index.

**Results:**

Higher genetically predicted SBP were associated with higher odds of preeclampsia or eclampsia [OR 1.81, 95% confidence interval (CI) 1.68–1.96, *P* = 5.45 × 10^−49^], preterm birth (OR 1.09, 95% CI 1.03–1.16, *P* = 0.005) and placental abruption (OR 1.33, 95% CI 1.05–1.68, *P* = 0.016). Higher genetically-predicted DBP was associated with preeclampsia or eclampsia (OR 2.54, 95% CI 2.21–2.92, *P* = 5.35 × 10^−40^). Higher genetically predicted PP was associated with preeclampsia or eclampsia (OR 1.68, 95% CI 1.47–1.92, *P* = 1.9 × 10^−14^) and preterm birth (OR 1.18, 95% CI 1.06–1.30, *P* = 0.002).

**Conclusion:**

This study provides genetic evidence to support causal associations of SBP, DBP and PP on multiple adverse outcomes of pregnancy. SBP and PP were associated with the broadest range of adverse outcomes, suggesting that optimized management of blood pressure, particularly SBP, is a key priority to improve feto-maternal health.

## INTRODUCTION

Hypertensive disorders of pregnancy are a major cause of maternal and fetal morbidity. The spectrum of hypertensive disorders of pregnancy encompasses chronic hypertension, gestational hypertension and preeclampsia [1]. Chronic hypertension either predates pregnancy or is diagnosed before 20 weeks’ gestation, whilst gestational hypertension arises *de novo* post-20 weeks’ gestation in the absence of proteinuria, biochemical or hematologic abnormalities [1]. Preeclampsia, at the more severe end of the spectrum, is characterized by hypertension occurring *de novo* after 20 weeks’ gestation with evidence of proteinuria, intrauterine growth restriction or end-organ damage. Multiple observational studies have demonstrated associations between maternal blood pressure prior to pregnancy and adverse maternal and fetal outcomes [2–4]. However, observational studies are liable to confounding and reverse causality. Even with careful adjustment for measured confounders, it is nearly impossible to accurately mirror the complex panoply of biological, behavioral and psychosocial factors that factor into the interplay between obstetric and cardiovascular risk. This is especially true for outcomes such as preterm birth and preeclampsia, where the pathophysiology remains relatively poorly understood. Improving our understanding of determinants of adverse feto-maternal outcomes in pregnancy is a public health priority, which may help improve risk stratification and targeted care provision to ultimately improve obstetric outcome.

Mendelian randomization leverages genetic variants as proxies for exposures in instrumental variable analysis to study exposure-outcome relationships. Given the independent and random distribution of genetic variants at meiosis, the Mendelian randomization paradigm is less vulnerable to limitations of phenotypic observational study, namely confounding and reverse causality. Our group previously applied the Mendelian randomization framework to provide genetic evidence for the causal effect of SBP, alongside other cardiometabolic traits, on preeclampsia or eclampsia risk [5]. We also demonstrated that SBP is associated with reduced birthweight independently of preeclampsia or eclampsia [5]. Further investigation of drug-target perturbation verified safety and efficacy profiles of genetically proxied beta-adrenergic antagonism and calcium channel blockade on risk of preeclampsia and low birthweight [6], echoing recent large randomized controlled trials (RCTs) [7,8]. However, the causal relevance of blood pressure on other important obstetric outcomes, such as preterm birth and placental abruption, remains unexplored. In addition, no genetic data exist exploring the role of blood pressure indices other than SBP. These data are relevant, as isolated diastolic hypertension is commonest in the age range overlapping with the majority of pregnancies. NHANES III highlighted that isolated diastolic hypertension was the commonest hypertensive subtype in participants aged less than 40 years [9], with the Framingham Heart study also showing that young age was a predictor for isolated diastolic hypertension [10].

In light of this evidence gap, we extended our prior Mendelian randomization work [5], to explore the additional causal relevance of genetically predicted DBP and pulse pressure (PP), on a broader range of maternal and fetal outcomes, including preterm birth, placental abruption and hemorrhage in early pregnancy. We thus utilized Mendelian randomization to explore the causal relevance of SBP, DBP and PP on adverse outcomes of pregnancy, including preeclampsia or eclampsia, preterm birth, placental abruption and hemorrhage in early pregnancy.

## MATERIALS AND METHODS

### Ethical approval, data availability and reporting

**FIGURE 1:**
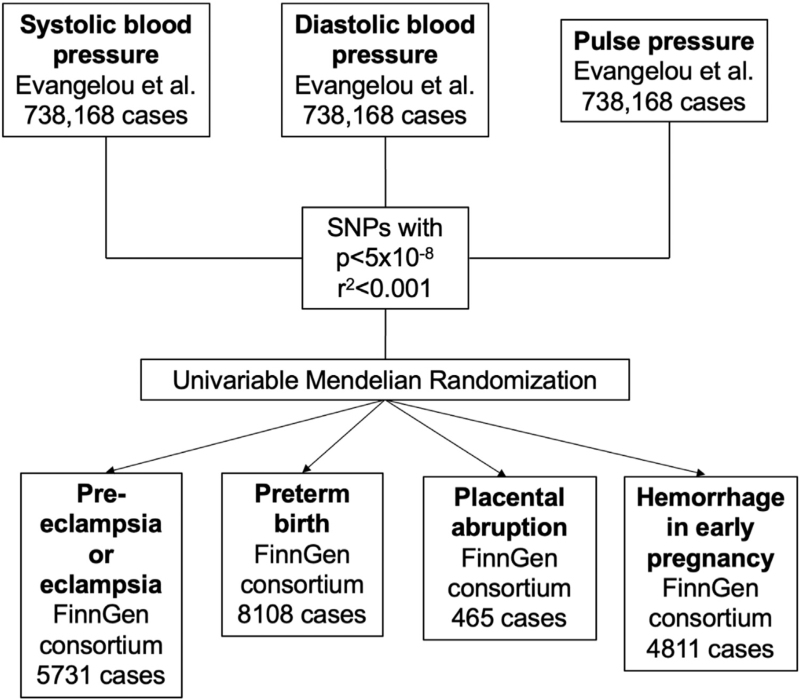
Data acquisition and analysis flowchart. SNPs, single nucleotide polymorphisms.

All data used in this study are openly available and appropriately cited. All original studies obtained relevant participant consent and ethical approval. This study is reported on the basis of recommendations by the STROBE-MR Guidelines [11]. A flowchart detailing the design of this study is provided in Fig. 1.

### Data sources

Instrumental variants for the exposures of SBP, DBP and PP were extracted from a sex-adjusted genome-wide association study (GWAS) on 738 168 individuals [12]. Instrumental SNPs for each exposure were selected if they were associated with the exposure at genome-wide significance level (*P* < 5 × 10^−8^). Only uncorrelated variants were retained, with pair-wise linkage disequilibrium with *r*^2^ less than 0.001. Clumping was performed using the TwoSample MR package in R [13].

The genetic association estimates of the instrumental variants with outcomes were extracted from summary statistics of GWAS analyses in FinnGen Round 7 female participants (*n*_total_ = 392 423, *n*_women_ = 219 462) [14]. These included 5731 cases for preeclampsia or eclampsia, 8108 cases for preterm birth, 465 cases for placental abruption and 4811 cases for hemorrhage in early pregnancy. Analyses were restricted to women for all outcomes. Definitions for all obstetric endpoints are based on the WHO International Statistical Classification of Diseases and Related Health Problems 10th Revision (ICD-10) codes. Details of relevant ICD-10 codes are provided in Table S1.

Detailed summaries of population characteristics for each of these cohorts are available in the original publications. A brief overview of the included studies is provided in Table S2. Only variants for which genetic association estimates were available for the exposure and outcome under investigation were considered in the analysis.

### Statistical analysis

Inverse-variance weighted Mendelian randomization with multiplicative random effects formed the primary analysis method for all models, to estimate the effect of genetically predicted SBP, DBP and PP on risk of adverse outcome of pregnancy. Results are presented as odds ratios (ORs) and 95% confidence intervals (95% CIs) per 10 mmHg higher genetically predicted SBP, DBP or PP. Sensitivity analyses using weighted median Mendelian randomization [15] and the MR-Egger [16] intercept test were used to assess the consistency of results using methods more robust to directional pleiotropy.

To account for multiple comparisons across the three primary exposures increasing the risk of type I statistical errors, the Bonferroni correction was applied. α was set at 0.0167 (0.05/3), *P* values less than 0.0167 were considered statistically significant. All statistical analyses were performed using the MendelianRandomisation package [17] in R version 4.2.1.

## RESULTS

### Diastolic blood pressure

Higher genetically predicted SBP was associated with higher odds of preeclampsia or eclampsia (OR 1.81, 95% CI 1.68–1.96, *P* = 5.45 × 10^−49^), consistent with previously reported results. In addition, higher genetically predicted SBP was associated with higher odds of preterm birth (OR 1.09, 95% CI 1.03–1.16, *P* = 0.005) and placental abruption (OR 1.33, 95% CI 1.05–1.68, *P* = 0.016).

**FIGURE 2:**
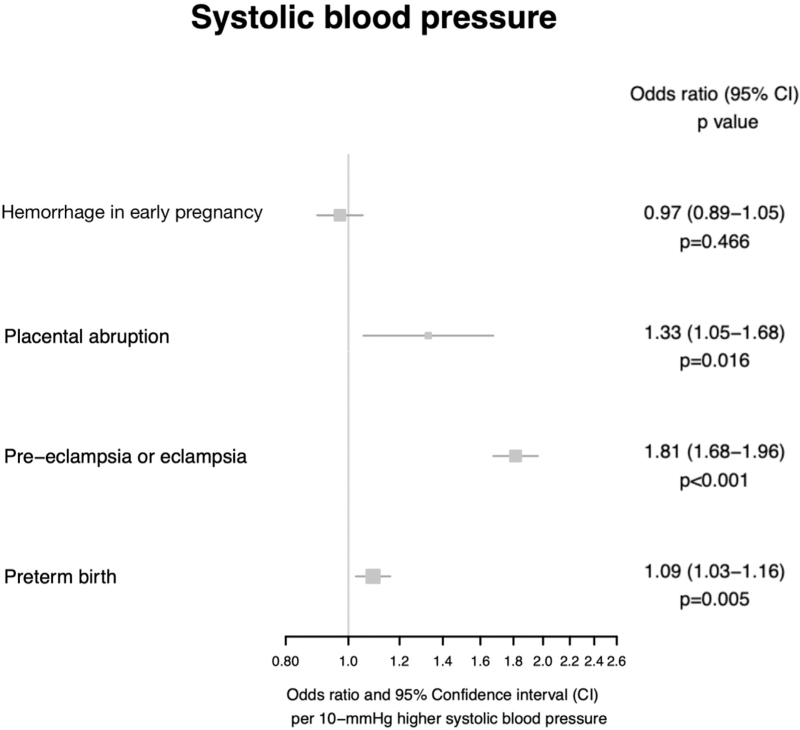
Mendelian randomization estimates displaying the effect of genetically predicted SBP on adverse outcomes of pregnancy.

There was no association between genetically predicted SBP and hemorrhage in early pregnancy (OR 0.97, 95% CI 0.89–1.05, *P* = 0.466). All Mendelian randomization estimates for SBP and adverse outcomes of pregnancy are displayed in Fig. 2.

### Systolic blood pressure

Higher genetically predicted DBP was associated with higher odds of preeclampsia or eclampsia (OR 2.54, 95% CI 2.21–2.92, *P* = 5.35 × 10^−40^).

**FIGURE 3:**
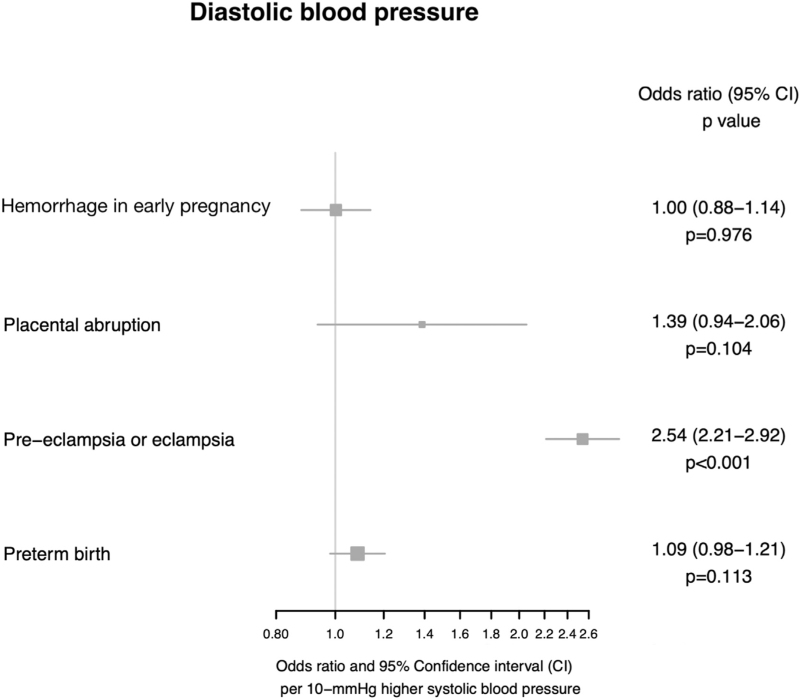
Mendelian randomization estimates displaying the effect of genetically predicted DBP on adverse outcomes of pregnancy.

There was no association between genetically-predicted DBP and preterm birth (OR 1.09, 95% CI 0.98–1.21, *P* = 0.113), placental abruption (OR 1.39, 95% CI 0.94–2.06, *P* = 0.104) or hemorrhage in early pregnancy (OR 1.002, 95% CI 0.88–1.14, *P* = 0.976). All Mendelian randomization estimates for DBP and adverse outcomes of pregnancy are displayed in Fig. 3.

### Pulse pressure

Higher genetically predicted PP was associated with higher odds of preeclampsia or eclampsia (OR 1.68, 95% CI 1.47–1.92, *P* = 1.90 × 10^−14^) and preterm birth (OR 1.18, 95% CI 1.06–1.30, *P* = 0.002).

**FIGURE 4:**
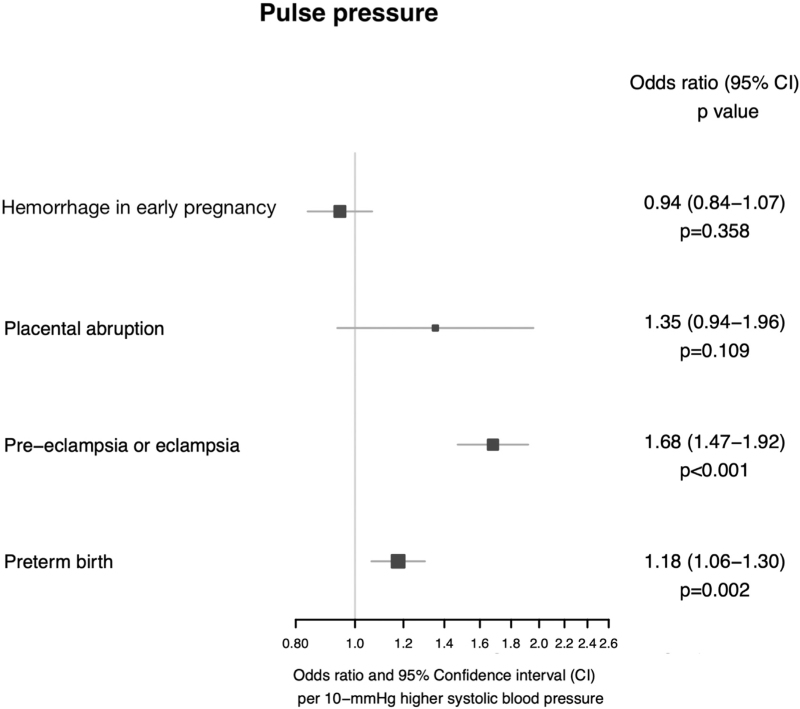
Mendelian randomization estimates displaying the effect of genetically predicted pulse pressure on adverse outcomes of pregnancy.

Higher genetically predicted PP was not associated with placental abruption (OR 1.35, 95% CI 0.94–1.96, *P* = 0.109), or hemorrhage in early pregnancy (OR 0.94, 95% CI 0.84–1.07, *P* = 0.358). All Mendelian randomization estimates for PP and adverse outcomes of pregnancy are displayed in Fig. 4.

### Sensitivity analyses

The results were largely consistent following sensitivity analyses, as displayed in Table S3.

## DISCUSSION

This study provides genetic evidence to support a causal role of higher genetically predicted SBP, DBP and PP on multiple adverse outcomes of pregnancy, including preeclampsia, preterm birth and placental abruption. The results have multiple important implications. First, they support recent clinical trial evidence that has shown improved outcomes of pregnancy with strict blood pressure control in women with gestational hypertension. Second, they identify key adverse outcomes associated with higher blood pressure that are of clinical relevance for risk stratification and surveillance in at-risk pregnancies. Finally, they support a causal relationship between all three blood pressure indices and adverse outcomes, highlighting the requirement for ongoing research aimed at carefully outlining blood pressure targets to aim for in different clinical situations in order to minimize risk of adverse outcomes.

### Preeclampsia and eclampsia

We previously reported the association between genetically predicted SBP with preeclampsia and eclampsia [5]. In this study, through our extended analyses, we showed that genetically predicted DBP and PP are also associated with risk of preeclampsia and eclampsia. This is consistent with multiple prior observational studies [18–22] and an observational meta-analysis [23]. Elevated DBP has been shown to be an independent predictor of preeclampsia in a large, health primiparous cohort [20] and DBP increases of at least 15 mmHg have been shown to be 92% sensitive for a diagnosis of preeclampsia [24]. Likewise, PP at 7–15 weeks has been shown to be significantly elevated in women who subsequently developed preeclampsia [21]. This study augments the previous literature by providing genetic evidence supporting a causal nature of this association. There are a number of potential mechanisms behind this. First, spiral artery formation might be impaired by a higher maternal blood pressure, purely through hemodynamic effects of the increased pressure and the severity of resultant vascular damage [25]. Alternatively, the alteration in the milieu of circulating vasoactive mediators known to occur secondary to hypertension might directly or indirectly impair spiral artery formation and effective placentation, leading to downstream risk of preeclampsia [26,27]. The subsequent failure of cytotrophoblasts to deeply invade the spiral arteries is thought to be related to failure to adopt an endothelial phenotype, with lack of expression of vascular adhesion molecules [28]. Instead of the physiological remodelling of decidual arteries into high-capacitance and low resistance vessels, hypertension contributes to development of constricted, high-resistance vessels that are inadequate for provision of oxygen and nutrients to the placenta and developing fetus [26]. Thus, the consequent histopathologic hallmark of defective placentation is decidual vasculopathy, which consists of features such as muscular hypertrophy, endothelial thickening, vascular thromboses and ectasia, vascular wall fibrinoid necrosis and atherosis [25]. Although this is often related to de-novo preeclampsia or that which is superimposed on chronic hypertension, there is evidence that features of decidual vasculopathy are also present in patients with gestational [29] and chronic hypertension [30].

### Preterm birth

In this study, genetically predicted SBP and PP were associated with increased odds of preterm birth. This is in line with previous literature. High baseline PP at 12 to 19 weeks’ gestation has been demonstrated to be a risk factor for early spontaneous preterm birth in normotensive nulliparous women, and rises in SBP and DBP between baseline and mid-third trimester values associated with an increased risk of early and late spontaneous preterm births in a dose--response manner [31] especially in women with obesity [32]. A plausible mechanism for this finding is that raised hypertensive indices increase preeclampsia risk: term preeclampsia in a first pregnancy is a major risk factor for subsequent preterm birth with or without recurrent preeclampsia [33]. Overall, elevated SBP and PP should alert clinicians to the possibility of pregnancies at a higher risk of preterm delivery and trigger appropriate peripartum care.

### Placental abruption

The association between higher genetically predicted SBP and placental abruption is supported by a prior observational meta-analysis of 54 studies [3] and a recent analysis linking U.S. natality and fetal death data in over 30 million pregnancies [34]. Together with the results of our study, these support the hypothesis that vascular and ischemic placental diseases, including abruption and preeclampsia, could be connected by a unifying cause [34]. From a mechanistic perspective, it is biologically plausible that higher blood pressure on the maternal side might encourage both the initiation and propagation of placental abruption, especially in the setting of increased resistance in the placental circulation. Given the life-threatening nature of this outcome, the results of this study suggest further work to assess the role of tight blood pressure control in women at risk for abruption.

### Hemorrhage in early pregnancy

This study did not find evidence of a relationship between genetically predicted hypertensive indices and increased odds of hemorrhage in early pregnancy. Hemorrhage in early pregnancy is a significant source of maternal anxiety and estimates of bleeding prevalence in early pregnancy range from 7 to 24% [35], and is associated with a higher risk of preterm delivery, antepartum hemorrhage, placenta previa and preterm premature rupture of membranes [36]. In the present study, we examined this outcome because it has been suggested that a mechanism behind it includes defective placentation, characterized by thinner and more fragmented trophoblast shells, and reduced cytotrophoblast invasion of spiral arterioles [36]. The lack of association in this study might reflect a lack of true underlying biological association with a blood pressure-related cause, given that other causes such as infection, inflammation, ectopic pregnancies and chorionic/cervical neoplasia may cause injury to the decidual vasculature or reproductive tract. An alternative explanation might relate to insufficient power in the instrumented SNPs. For this reason, the null result should not be interpreted as final evidence of lack of an association.

### Strengths and limitations

For the first time, this Mendelian randomization study provides genetic evidence for the causal effects of hypertensive indices on a variety of adverse outcomes of pregnancy, including novel data on DBP and PP. Key to the present analysis is reduced liability to confounding. As gametes are independently and randomly distributed at conception, the Mendelian randomization paradigm is analogous to randomization to higher or lower blood pressure targets in an RCT. Furthermore, as with RCTs, reverse causality is minimized because inheritance of genetic variants temporally precedes outcomes.

Limitations of this study exist. First, we were unable to parse the role of genetically predicted hypertensive indices on the potentially important subtypes of early versus late-onset preeclampsia, or early versus late preterm birth as the requisite genetic association data did not exist. Second, the Bonferroni correction is a conservative approach to the multiple comparisons problem with a greater risk of failure to reject false null hypotheses, thereby increasing the risk of a type II statistical error. In the same vein however, the associations reported are likely to be robust and not the result of false positives, given the high level of statistical significance demonstrated. Third, the data sources for this study concentrated in European ancestry individuals. By including an ethnically homogenous population, we minimized the possibility of confounding by population stratification; however, the results may not be fully generalizable to all ethnicities. Replication on more ancestrally diverse cohorts when data become available is salient, given the high prevalence of chronic hypertension in many non-European ethnicities [37].

### Clinical relevance

The results of our study stress the fact that optimal blood pressure management, for all three blood pressure indices, is likely to play a key role in optimization of maternal and fetal outcomes in pregnancy. Given the highlighted importance of blood pressure management, it is important to mention that considerable work remains to be done in outlining exact, evidence-based blood pressure targets in pregnancy across a spectrum of different clinical situations. Recently, the practice-changing Chronic Hypertension and Pregnancy (CHAP) randomized trial demonstrated that a lower threshold for treating chronic hypertension in pregnancy (140/90 mmHg) resulted in an 18% reduction in a primary composite outcome of preeclampsia, preterm birth, placental abruption or fetal/neonatal death, compared with a treatment threshold of 160/105 mmHg or greater [8]. The clinically important difference highlighted in this study emphasizes the clear unmet need for improved clarity in definition of blood pressure thresholds for treatment in pregnancy, and the results of our present study, causally linking blood pressure traits with adverse outcomes, further stress this. Optimization of blood pressure also bears relevance for future maternal health beyond the antenatal and peripartum periods. A systematic review and meta-analysis of 73 observational studies demonstrated that women with preeclampsia had around two-fold increased risk of cardiovascular and cerebrovascular disease, in addition to over double the risk of cardiovascular death compared with those with previous normotensive pregnancy [38]. Similarly, in parous participants without prior cardiovascular disease enrolled within the Nurses’ Health Study II, women with hypertensive disorders of pregnancy in their first pregnancy had a 63% higher hazard of later cardiovascular disease compared with normotensive pregnancies [39]. The increase in cardiovascular disease was consistent in women with gestational hypertension or preeclampsia, though preeclampsia conferred a larger hazard. We recently applied Mendelian randomization to corroborate these observational data and revealed that genetically predicted hypertensive disorders of pregnancy likely causally associate with risk of coronary artery disease and stroke [40]. Ongoing trials aimed at defining clear optimal antenatal blood pressure targets for different clinical situations (e.g. based on preexisting hypertension, preeclampsia, obstetric risk level) are a key priority for the improvement of both obstetric outcomes and the lifelong health of parous women.

In conclusion, this study illuminates the effects of genetically predicted hypertensive indices, namely SBP, DBP and PP, on various adverse outcomes of pregnancy. Higher hypertensive indices may be useful to identify women whose pregnancies might benefit from enhanced surveillance and eventually therapeutic intervention. The results stress the key importance of blood pressure management by highlighting the importance of all three blood pressure indices as major direct drivers of multiple adverse outcomes of pregnancy. Further research elucidating optimal blood pressure targets in different clinical situations is a key priority.

## ACKNOWLEDGEMENTS

The authors would like to acknowledge the participants and investigators of the FinnGen project, UK Biobank and the International Consortium of Blood Pressure Genome Wide Association Studies.

This study was supported by Imperial National Institute for Health Research (NIHR) Biomedical Research Centre for RKR, NIHR Academic Clinical Fellowship for M.A., NIHR Cambridge Biomedical Research Centre BRC-1215-20014 for S.B., Imperial NIHR Biomedical Research Centre funding for F.S.N.

British Heart Foundation (RG/F/22/110078 and RE/18/4/34215 for F.S.N.).

All other authors report no relevant funding.

This work has been accepted for presentation at the American College of Cardiology Annual Scientific Sessions 2023.

### Conflicts of interest

The authors report no relevant disclosures or conflicts of interest.

## Supplementary Material

Supplemental Digital Content
