# Supplementary material for: Maternal hypertensive traits and adverse outcome in pregnancy: a Mendelian randomization study
Source: J Hypertens. 2023 Jul 5;41(9):1438–45. doi: 10.1097/HJH.0000000000003486 (PMC10399932; doi:10.1097/HJH.0000000000003486)
Supplement: Supplemental Digital Content [file jhype-41-1438-s001.docx]

**Supplementary Data**

**Maternal hemodynamic traits and adverse outcome in pregnancy: a Mendelian randomization study**

Maddalena Ardissino*^1,2^, Rohin K. Reddy*^1^, Eric A. W. Slob^3-5^, Jack Griffiths^6^, Joanna Girling^7^, Fu Siong Ng^1,7^

*Co-first authors with equal contribution

^1^National Heart and Lung Institute, Imperial College London, London, UK

^2^Royal Papworth Hospital, Cambridge Biomedical Campus, Cambridge, UK

^3^MRC Biostatistics Unit, School of Clinical Medicine, University of Cambridge, Cambridge, UK

^4^Department of Applied Economics, Erasmus School of Economics, Erasmus University Rotterdam, Rotterdam, The Netherlands

^5^Erasmus University Rotterdam Institute for Behavior and Biology, Erasmus University Rotterdam, Rotterdam, The Netherlands

^6^Royal Brompton Hospital, Guy’s and St Thomas’ NHS Foundation Trust, London, UK

^7^West Middlesex Hospital, Chelsea and Westminster Hospital NHS Foundation Trust, London, UK

**Contents**

**Table S1.** Case and control definitions for obstetric outcomes used within this study, based on the World Health Organization International Statistical Classification of Diseases and Related Health Problems 10th Revision (ICD-10) codes and the FinnGen project.………… ………....………………………………………….3

**Table S2.** Demographic information for the genome-wide association studies used as genetic association data sources……………………………..…………………... ……….………………………...…………………….5

**Table S3.** Mendelian randomization (MR) sensitivity analyses for effects of genetically-predicted hypertensive traits on adverse outcomes of pregnancy, using weighted median MR and MR-Egger models…………………………………………………………………………………………………………………..6

**Table S1** – Case and control definitions for obstetric outcomes used within this study, based on the World Health Organization International Statistical Classification of Diseases and Related Health Problems 10th Revision (ICD-10) codes and the FinnGen project.

| Outcome | ICD-10 code(s) |
| --- | --- |
| Pre-eclampsia or eclampsia | **O11 Pre-eclampsia superimposed on chronic hypertension**  *Including:*   - Conditions in O10 (*Pre-existing hypertension complicating pregnancy, childbirth and the puerperium*) complicated by pre-eclampsia - Pre-eclampsia superimposed on hypertension NOS or pre-existing hypertension   **O14 Pre-eclampsia**  *Including:*   - O14.0 Mild to moderate pre-eclampsia - O14.1 Severe pre-eclampsia - O14.2 HELLP syndrome (combination of hemolysis, elevated liver enzymes and low platelet count) - O14.9 Pre-eclampsia, unspecified   *Excluding:*   - Superimposed pre-eclampsia (O11)   **O15 Eclampsia**  *Including:*   - Convulsions following conditions in O10-O14 and O16 eclampsia with pregnancy-induced or pre-existing hypertension - O15.0 Eclampsia in pregnancy - O15.1 Eclampsia in labour - O15.2 Eclampsia in the puerperium - O15.9 Eclampsia, unspecified as to time period - Eclampsia NOS   **Control definition**   - Individuals that were not cases - Women without oedema, proteinuria and hypertensive disorders in pregnancy, childbirth and the puerperium (FinnGen code = O15_OEDEM_PROTUR_HYPERT) |
| Preterm birth | **O60 Preterm labour and delivery**  *Including:*   - Onset (spontaneous) of labour before 37 completed weeks of gestation - O60.1 Preterm spontaneous labour with preterm delivery: Preterm labour with delivery NOS, Preterm spontaneous labour with preterm delivery by caesarean section - O60.2 Preterm spontaneous labour with term delivery: Preterm spontaneous labour with term delivery by caesarean section - O60.3 Preterm delivery without spontaneous labour: Preterm delivery by caesarean section without spontaneous labour or induction   **Control definition**   - Individuals that were not cases - Women without complications of labour and delivery (FinnGen code = O15_COMPLIC_LAB_DELIV) |
| Placental abruption | **O45 Premature separation of placenta [abruptio placentae]**   - O45.0 Premature separation of placenta with coagulation defect: Abruptio placentae with (excessive) hemorrhage associated with afibrinogenaemia, disseminated intravascular coagulation, hyperfibrinolysis, hypofibrinogenaemia - O45.8 Other premature separation of placenta - O45.9 Premature separation of placenta, unspecified: Abruptio placentae NOS   **Control definition**   - Individuals that were not cases - Women without complications relating to maternal care related to the fetus and amniotic cavity and possible delivery problems (FinnGen code = O15_MATERN_CARE) |
| Hemorrhage in early pregnancy | **O20 Hemorrhage in early pregnancy**  *Including:*   - O20.0 Threatened abortion: Hemorrhage specified as due to threatened abortion - O20.8 Other hemorrhage in early pregnancy - O20.9 Hemorrhage in early pregnancy, unspecified   *Excluding:*   - Pregnancy with abortive outcome (O00-O08)   **Control definition**   - Individuals that were not cases - Women without maternal disorders predominantly related to pregnancy (FinnGen code = O15_PREG_OTHER_MAT_DISORD) |

**Table S2** – Demographic information for the genome-wide association studies used as genetic association data sources. SD = standard deviation.

| Variable | Study author | Number of cases and controls (n_cases_, n_controls_) | Ethnic ancestry | Unit | PMID/link |
| --- | --- | --- | --- | --- | --- |
| Exposures | | | | | |
| Systolic blood pressure | Evangelou  et al. | 738,168 | Mixed | 1-SD mmHg | 30224653 |
| Diastolic blood pressure |  |  |  |  |  |
| Pulse pressure |  |  |  |  |  |
| Outcomes | | | | | |
| Pre-eclampsia or eclampsia | FinnGen Round 7 | 5731, 160670 | European | Odds ratio | <https://finngen.gitbook.io/documentation> |
| Preterm birth |  | 8108, 135806 |  |  |  |
| Placental abruption |  | 465, 142734 |  |  |  |
| Hemorrhage in early pregnancy |  | 4811, 152785 |  |  |  |

**Table S3** – Mendelian randomization (MR) sensitivity analyses for effects of genetically-predicted hypertensive traits on adverse outcomes of pregnancy, using weighted median MR and MR-Egger models. CI = confidence interval.

| Exposure | Outcome | Method | Beta coef | Lower 95% CI | Upper 95% CI | P-value |
| --- | --- | --- | --- | --- | --- | --- |
| Systolic blood pressure  (10-unit increase, mmHg) | Pre-eclampsia or eclampsia | Weighted median | 0.06 | 0.04 | 0.07 | 1.40x10^-21^ |
|  |  | MR-Egger | 0.08 | 0.06 | 0.10 | 1.89x10^-15^ |
|  |  |  |  |  | intercept | 0.008 |
|  | Pre-term birth | Weighted median | 3.0x10^-3^ | -6.4x10^-3^ | 0.01 | 0.532 |
|  |  | MR-Egger | 0.01 | -0.01 | 0.03 | 0.185 |
|  |  |  |  |  | intercept | 0.800 |
|  | Placental abruption | Weighted median | 0.02 | -0.01 | 0.06 | 0.241 |
|  |  | MR-Egger | 0.06 | -9.6x10^-4^ | 0.09 | 0.047 |
|  |  |  |  |  | intercept | 0.259 |
|  | Hemorrhage in early pregnancy | Weighted median | -3x10^-3^ | -0.02 | 8x10^-3^ | 0.554 |
|  |  | MR-Egger | 4.6x10^-3^ | -0.03 | 0.02 | 0.660 |
|  |  |  |  |  | intercept | 0.866 |
| Diastolic blood pressure  (10-unit increase, mmHg) | Pre-eclampsia or eclampsia | Weighted median | 0.09 | 0.07 | 0.11 | 8.27x10^-21^ |
|  |  | MR-Egger | 0.13 | 0.10 | 0.17 | 7.61x10^-14^ |
|  |  |  |  |  | intercept | 0.010 |
|  | Pre-term birth | Weighted median | 1.9x10^-3^ | -0.01 | 0.02 | 0.809 |
|  |  | MR-Egger | 0.01 | -0.02 | 0.03 | 0.541 |
|  |  |  |  |  | intercept | 0.977 |
|  | Placental abruption | Weighted median | 0.04 | -0.02 | 0.10 | 0.226 |
|  |  | MR-Egger | 0.09 | -0.01 | 0.19 | 0.072 |
|  |  |  |  |  | intercept | 0.210 |
|  | Hemorrhage in early pregnancy | Weighted median | -4.6x10^-3^ | -0.02 | 0.02 | 0.654 |
|  |  | MR-Egger | -0.03 | -0.06 | 7.6x10^-3^ | 0.135 |
|  |  |  |  |  | intercept | 0.099 |
| Pulse pressure  (10-unit increase, mmHg) | Pre-eclampsia or eclampsia | Weighted median | 0.04 | 0.02 | 0.06 | 1.93x10^-5^ |
|  |  | MR-Egger | 0.06 | 0.03 | 0.09 | 3.6x10^-5^ |
|  |  |  |  |  | intercept | 0.558 |
|  | Pre-term birth | Weighted median | 4.4x10^-3^ | -0.01 | 0.02 | 0.553 |
|  |  | MR-Egger | 0.01 | -0.02 | 0.03 | 0.579 |
|  |  |  |  |  | intercept | 0.441 |
|  | Placental abruption | Weighted median | 0.04 | -0.02 | 0.09 | 0.200 |
|  |  | MR-Egger | 0.06 | 9x10^-4^ | 0.12 | 0.047 |
|  |  |  |  |  | intercept | 0.484 |
|  | Hemorrhage in early pregnancy | Weighted median | -0.01 | -0.03 | 6.8x10^-3^ | 0.223 |
|  |  | MR-Egger | 0.01 | -0.02 | 0.04 | 0.589 |
|  |  |  |  |  | intercept | 0.322 |
